# Advancing Patient Care: The Role of Nutrition in Immune-Mediated Diseases, Spanning from Cancer to Autoimmunity

**DOI:** 10.3390/nu17233632

**Published:** 2025-11-21

**Authors:** Elena Niccolai

**Affiliations:** Department of Experimental and Clinical Medicine, University of Florence, Largo Brambilla 3, 50134 Florence, Italy; elena.niccolai@unifi.it

The interrelationship between nutrition and immune function has moved from a niche interest into a central axis of investigation for immune-mediated diseases. As immunology, metabolism, microbiome research, and nutritional science increasingly intersect, it has become clear that dietary habits not only influence susceptibility to infection but also shape the development, progression, and therapeutic responsiveness of a wide spectrum of immune-mediated disorders, from malignancies to autoimmune and chronic inflammatory diseases.

For decades, therapeutic approaches to immune-mediated conditions—whether inflammatory, autoimmune, or neoplastic—have concentrated on pharmacologic immunomodulation, biologic therapies, and cellular interventions such as immune checkpoint inhibitors and adoptive T-cell transfer. Nutrition, by contrast, was largely relegated to the background, considered supportive rather than central to disease management. That view is now changing. Increasing evidence indicates that nutritional status, dietary patterns, and the composition of micro- and macronutrients, as well as the intricate interactions between diet and the gut microbiome, can profoundly modulate immune cell populations, cytokine networks, and immunometabolism, thereby influencing disease initiation, trajectory, and treatment outcomes [1,2,3].

This shift is particularly relevant at a time when the incidence and burden of immune-mediated diseases are rising globally [4]. Autoimmune disorders, inflammatory bowel disease, psoriasis, and cancers with an immunologic component all contribute to a growing health challenge. Integrating nutrition into patient care offers the prospect of precision immunonutrition—tailoring dietary interventions and nutritional support alongside immune-targeting therapies to enhance clinical efficacy and overall well-being.

Mechanistic studies have elucidated how key nutrients—including vitamins A, D, E, C, and the B complex, along with trace minerals such as zinc, selenium, and iron, and bioactive compounds like polyunsaturated fatty acids, fibre, and polyphenols—exert immunomodulatory effects [3,5,6,7]. These micronutrients regulate lymphoid tissue development, immune cell differentiation, and systemic inflammation, as elegantly summarized by Méndez López and colleagues [8]. In cancer biology, the “nutrition–microbiome–immune” triangle has become a pivotal area of investigation. Dietary fibre, metabolized by gut microbes into short-chain fatty acids (SCFAs), activates G-protein-coupled receptors on immune cells and modulates histone deacetylase activity, thereby shaping T-cell and natural killer (NK) cell functions within the tumour microenvironment [9,10]. The integration of immunonutrition and microbiota modulation into oncologic care has been recently emphasized by Martinelli et al., who reviewed synergistic strategies for gastrointestinal (GI) cancer management, showing how specific immunonutrients and microbiota-targeted interventions can improve nutritional status, postoperative recovery, and response to therapies by mitigating inflammation and enhancing immune competence [11]. This growing body of evidence reinforces the notion that diet–microbiome interactions are not only preventive but also therapeutic tools capable of modulating tumour immunology and treatment responsiveness.

Likewise, in autoimmune and inflammatory diseases, the link between diet, gut barrier integrity, microbiome composition, and immune dysregulation is increasingly well-established [12,13,14]. Together, these findings highlight the therapeutic potential of dietary modulation of immunometabolism.

From a clinical standpoint, the implications of this evolving understanding are substantial. Nutritional strategies can play a preventive role in individuals at high risk for immune-mediated disorders—those with familial autoimmune disease, chronic inflammation, or pre-neoplastic immune dysregulation—by maintaining immune equilibrium and limiting the pro-inflammatory milieu that predisposes to disease. In established disease, nutrition can be leveraged to enhance immune effector functions, sustain remission, and mitigate treatment toxicities. Specific dietary patterns, such as the Mediterranean diet rich in anti-inflammatory and antioxidant constituents, have shown encouraging results in various inflammatory and autoimmune contexts [15]. In oncology, nutritional interventions including ketogenic, fasting-mimicking, and high-fibre diets are being explored for their potential to improve responsiveness to immune-based therapies [10].

Evidence supporting the clinical impact of nutritional care continues to grow. Cotogni et al. demonstrated that in advanced cancer, the early initiation of home parenteral nutrition (HPN), when aligned with European guidelines, significantly improved global quality of life even in the last two months of life, underscoring that nutritional support can enhance not only survival but also end-of-life well-being [16]. In addition, lifestyle and nutrition extend their influence beyond prevention to the phase of survivorship. Li et al. showed that colorectal cancer patients maintaining healthy post-operative habits—adequate fruit and vegetable intake, regular physical activity, good sleep quality, and normal body weight—experienced significantly better health-related quality of life during follow-up, reinforcing the importance of embedding nutritional and behavioural care into recovery and long-term management [17]. Importantly, Lo Buglio et al. reported that adherence to the Mediterranean diet mitigated systemic inflammation and reduced hospital stays among frail elderly patients, with clear correlations between nutritional status, inflammatory biomarkers such as CRP, and clinical outcomes [18].

The reach of nutritional immunomodulation now extends into neuroinflammatory and neurodegenerative diseases. Cuffaro et al. reviewed the evidence linking metabolic alterations and gut microbiota dysbiosis to the onset and progression of amyotrophic lateral sclerosis (ALS), proposing personalized nutritional and microbiota-targeted interventions—including antioxidant-rich, anti-inflammatory diets, and the use of probiotics, prebiotics, and postbiotics—to restore gut–brain axis integrity and mitigate neuroinflammation [19]. This represents an important step toward precision immunonutrition in neurodegenerative contexts. Likewise, Bento da Nave and colleagues analyzed randomized clinical trials investigating polyunsaturated fatty acid (PUFA) supplementation in Sjögren’s syndrome, observing potential benefits in symptomatology and inflammatory parameters, while emphasizing the need for further high-quality trials. These findings point toward the potential for targeted lipid-based interventions to modify disease trajectories in autoimmune settings [20].

Taken together, these insights herald the era of precision immunonutrition, which seeks to align dietary interventions, bioactive nutrient supplementation, microbiome modulation, and metabolic support with the individual patient’s genetic, metabolic, and immunologic profile. The traditional “one-size-fits-all” approach to diet is giving way to personalized strategies designed to optimize immune resilience and therapeutic response. Yet, the transition from promising evidence to routine clinical application remains challenging. Many dietary intervention studies in immune-mediated inflammatory diseases continue to suffer from methodological constraints—heterogeneous dietary assessments, small sample sizes, short follow-up periods, and incomplete control of confounding factors. As Guerrero Aznar MD et al. have highlighted, the overall quality of evidence in meta-analyses assessing dietary composition remains low or critically low [15]. Moreover, mechanistic clarity is still incomplete: it is not yet fully understood how specific nutrients influence the balance between regulatory and effector T cells in humans, or how microbiome-derived metabolites integrate with diet to orchestrate systemic immune responses.

Translating these scientific insights into practice requires a more integrated framework for patient care. Nutrition must become a structural component of multidisciplinary management, with dietitians embedded within teams managing complex immune-mediated diseases. Effective dietary interventions must be feasible, sustainable, and synergistic with immunologic treatments, considering timing, potential nutrient–drug interactions, and patient adherence. Future progress will depend on the development of robust biomarkers—microbiome and metabolomic signatures, immunophenotyping tools, and predictive models linking dietary exposures to immune outcomes. Only through such integration can nutritional science fully enter the precision medicine paradigm and deliver on its promise to improve the lives of patients living with immune-mediated diseases.
